# Retraction: Effect and potential mechanism of *Lactobacillus plantarum* Q7 on hyperuricemia *in vitro* and *in vivo*

**DOI:** 10.3389/fnut.2024.1517030

**Published:** 2024-11-01

**Authors:** Frontiers Editorial Office

The journal retracts the July 2022 article cited above.

Following publication, concerns were raised regarding the duplicate publication of data in the article cited above in *Frontiers in Nutrition* and an article published in *Frontiers in Immunology* by Cao J, Liu Q, Hao H, Bu Y, Tian X, Wang T and Yi H (2022) Lactobacillus paracasei X11 Ameliorates Hyperuricemia and Modulates Gut Microbiota in Mice. *Front. Immunol*. 13:940228. doi: 10.3389/fimmu.2022.940228. An investigation was conducted in accordance with Frontiers' policies, which confirmed the concerns. Following the Committee on Publication Ethics guidelines, this article is retracted.

The authors received a communication regarding the retraction and had a chance to respond. This communication is recorded by the publisher.

